# IGF2BP1 promotes SRF-dependent transcription in cancer in a m^6^A- and miRNA-dependent manner

**DOI:** 10.1093/nar/gky1012

**Published:** 2018-10-29

**Authors:** Simon Müller, Markus Glaß, Anurag K Singh, Jacob Haase, Nadine Bley, Tommy Fuchs, Marcell Lederer, Andreas Dahl, Huilin Huang, Jianjun Chen, Guido Posern, Stefan Hüttelmaier

**Affiliations:** 1 Institute of Molecular Medicine, Section for Molecular Cell Biology, Faculty of Medicine, Martin Luther University Halle-Wittenberg, Charles Tanford protein center, Kurt-Mothes-Str. 3a, 06120 Halle, Germany; 2 Institute for Physiological Chemistry, Medical Faculty, Martin Luther University Halle-Wittenberg, 06114 Halle (Saale), Germany; 3 Deep Sequencing Group, Center for Molecular and Cellular Bioengineering, Technische Universität Dresden, Tatzberg 47/49, 01307 Dresden; 4 Department of Systems Biology, City of Hope, Monrovia, CA 91016, USA; 5 Department of Cancer Biology, University of Cincinnati College of Medicine, Cincinnati, OH 45219, USA

## Abstract

The oncofetal mRNA-binding protein IGF2BP1 and the transcriptional regulator SRF modulate gene expression in cancer. In cancer cells, we demonstrate that IGF2BP1 promotes the expression of SRF in a conserved and N^6^-methyladenosine (m^6^A)-dependent manner by impairing the miRNA-directed decay of the SRF mRNA. This results in enhanced SRF-dependent transcriptional activity and promotes tumor cell growth and invasion. At the post-transcriptional level, IGF2BP1 sustains the expression of various SRF-target genes. The majority of these SRF/IGF2BP1-enhanced genes, including PDLIM7 and FOXK1, show conserved upregulation with SRF and IGF2BP1 synthesis in cancer. PDLIM7 and FOXK1 promote tumor cell growth and were reported to enhance cell invasion. Consistently, 35 SRF/IGF2BP1-dependent genes showing conserved association with SRF and IGF2BP1 expression indicate a poor overall survival probability in ovarian, liver and lung cancer. In conclusion, these findings identify the SRF/IGF2BP1-, miRNome- and m^6^A-dependent control of gene expression as a conserved oncogenic driver network in cancer.

## INTRODUCTION

The mammalian IGF2 mRNA binding proteins (IGF2BPs; alias: VICKZ, CRD-BP, IMPs or ZBPs) family encompasses three RNA-binding proteins (RBPs) controlling the cytoplasmic fate of mRNAs in development, somatic cells and human diseases (1). Two members, IGF2BP1 and 3, are *bona fide* oncofetal proteins (1,2). They are abundant during development, expressed in some progenitor cells, barely observed in adult life but become upregulated or *de novo* synthesized in cancer (1,3–5). Recent studies indicate that IGF2BP1 has the most conserved ‘oncogenic’ role of the IGF2BP family in tumor-derived cells (6). The protein promotes a mesenchymal tumor cell phenotype characterized by altered actin dynamics, elevated migration, invasion, proliferation, self-renewal and anoikis resistance (7–9). Consistently, IGF2BP1 expression is associated with poor prognosis in various human cancers and the protein enhances the growth and metastasis of human tumor-derived cells in nude mice, as demonstrated for epithelial ovarian cancer (EOC) as well as hepatocellular carcinoma (HCC) derived tumor cells (6,10). This ‘oncogenic’ role of IGF2BP1 essentially relies on the impairment of mRNA decay. By associating with its target mRNAs, IGF2BP1 interferes with the degradation of target transcripts by endonucleases, as demonstrated for the MYC mRNA (11,12), or miRNA-directed decay, as shown for the vast majority of by now validated target mRNAs (6,9,13). Recent studies revealed that the association of IGF2BPs with target mRNAs, e.g. the MYC mRNA, is enhanced by the N^6^-methyladenosine (m^6^A) modification of target transcripts suggesting IGF2BPs as novel m^6^A-readers (14). Cross-linking immunoprecipitation (CLIP) analyses identified a plethora of candidate target mRNAs of IGF2BPs and revealed the 3′UTR as the mainly bound cis-element in associated transcripts (15–17). Although these studies indicate a substantial conservation of IGF2BP–mRNA association in tumor and stem cells, the phenotypic roles of IGF2BP homologs show a large variability in tumor cells derived from distinct cancers (6). The conserved phenotypic role of IGF2BP1 in tumor-derived cells suggests that the protein, in addition to promoting MYC synthesis, enhances additional oncogenic pathways not or barely affected by the other IGF2BP homologs.

In this study, we identify the SRF-encoding (serum response factor) mRNA as a conserved target mRNA of IGF2BP1 in cancer. SRF controls gene expression in concert with two classes of regulators: ternary complex factors (TCFs: ELK1, 3 and 4) and myocardin-related transcription factors (MRTFA and MRTFB) (18). Transcriptomic analyses revealed that SRF-MRTF driven transcription modulates the expression of genes involved in cytoskeletal regulation, cell adhesion, migration and invasion (19–21). Although partially overlapping, SRF/TCF-dependent gene expression mainly affects genes modulating proliferation and growth factor responsiveness (20,22). The SRF/MRTF-dependent control of gene expression essentially relies on RhoGTPase-signaling and actin dynamics modulating the subcellular localization and activity of MRTFs in transcription (23,24). Transcriptional control by SRF/TCFs is regulated by Mitogen-activated protein kinase-signaling (MAPK-signaling) (18,25). Thus, in concert with MRTFs and TCFs, SRF serves as a central hub modulating tumor cell migration, invasion and metastasis as well as proliferation and tumor growth in a signaling- and cytoskeleton-dependent manner (26–28). Notably, recent studies indicate that SRF destabilizes cell identity, promotes cellular reprogramming to pluripotency and when overexpressed in mice even enhances a metaplasia-like phenotype in the pancreas (29).

Here, we demonstrate that IGF2BP1 promotes SRF and SRF target genes at the post-transcriptional level suggesting it as a post-transcriptional enhancer of SRF itself as well as SRF-dependent gene expression in cancer cells. IGF2BP1 promotes SRF expression in a m^6^A-dependent manner by impairing the miRNA-directed downregulation of the SRF mRNA. In addition, IGF2BP1 enhances the expression of SRF-induced target genes at the post-transcriptional level. In cancer, the SRF-IGF2BP1 directed enhancement of gene expression promotes an ‘aggressive’ tumor cell phenotype and is associated with poor prognosis.

## MATERIALS AND METHODS

### Plasmids and cloning

Information on cloning strategies including vectors, oligonucleotides used for polymerase chain reaction (PCR) and restrictions sites are summarized in Supplementary Table ST5. All constructs were validated by sequencing.

### ChIP, RIP and RT-qPCR

Chromatin immunoprecipitation (ChIP) experiments were performed essentially as described previously (30). In brief, ∼2.5 × 10^7^ ES-2 cells were treated with formaldehyde, quenched and harvested in lysis buffer (10 mM 4-(2-hydroxyethyl)-1-piperazineethanesulfonic acid (HEPES), pH 7.9; 7.2 mM KOH; 150 mM KCl; 5 mM MgCl_2_; 0.5% NP-40; protease inhibitors). Nuclei were enriched by centrifugation and lysed in ChIP-buffer (50 mM Tris-Cl, pH 8.0; 10 mM ethylenediaminetetraacetic acid (EDTA); 1% sodium dodecyl sulphate (SDS); protease inhibitors) before chromatin was sheared by sonification. For ChIP, 25 μg of sheared chromatin was incubated with control (anti-IgG, Abcam ab171870) or anti-SRF (NEB 5147) antibodies overnight in dilution buffer (10 mM Tris-Cl, pH 8.0; 150 mM NaCl; 1 mM EDTA; 0.1% SDS; 1% Triton X-100; protease inhibitors). Upon extensive washing, chromatin was eluted in elution buffer (50 mM Tris-Cl, pH 8.0; 10 mM EDTA; 1% SDS), treated with Proteinase K and cross-linking was reversed overnight. DNA was finally eluted using the WIZARD^®^SV Gel & PCR Clean-Up System (Promega A9281) according to the manufacturer’s protocol and analyzed by quantitative real-time PCR (qPCR). RNA immunoprecipitation (RIP) and quantitative RT-PCR analyses were performed essentially as recently described (6). Primers are summarized in Supplementary Table ST5.

### Northern and western blotting

Northern blotting of small RNAs and semi-quantitative infrared western blotting were performed as recently described (9). Probes and antibodies are summarized in Supplementary Tables ST5 and ST7.

### Luciferase reporter assays

Promoter reporter assays were performed using the MRTF-specific 3.DA reporter (24) and a TCF-dependent reporter containing 500 bp of the murine Egr1 promoter cloned into pGL3 and the pRL-tk plasmid (kind gift from Bernd Knöll, Ulm University, Germany). Cells were retransfected with siRNAs using RNAiMax for 24 h before reporter transfection with polyethylenimine and harvested for analysis the following day, as described previously (31). Firefly luciferase activity was normalized to fluorescence of co-transfected EGFP (BMG Labtech Clariostar microplate reader) and was shown relative to the control siRNA transfection.

For the analysis of miRNAs targeting the SRF 3′UTR, 48-nt long regions of the SRF 3′UTR comprising predicted miRNA targeting sites (MTSs) were cloned 3′ to the firefly luciferase open reading frame (pmirGLO, Promega). Luciferase reporter analyses were performed as previously described (9). The activities of firefly and renilla luciferases were determined 48 h post-transfection by DualGLO (Promega) according to the manufacturer’s protocol. Reporters containing a minimal vector-encoded 3′UTR (empty) served as normalization controls.

### RNA sequencing and differential gene expression

Libraries for RNA-sequencing (RNA-seq) were essentially prepared according to the manufacturer’s protocols. For total RNA-seq, 1 μg of total RNA served as input for ribosomal RNA depletion using RiboCop v1.2 (Lexogen). The Ultra Directional RNA Library kit (NEB) was used for library generation. Sequencing was performed on an Illumina NextSeq 500 platform. For the generation of small RNA-seq libraries, 50 ng of total RNA served as input using the NEXTflex Small RNA Library Prep Kit v3 (Bio Scientific). Sequencing was performed on the Illumina HighSeq 2000 platform.

For RNA-seq data analyses, low quality read ends as well as remaining parts of sequencing adapters were clipped off using Cutadapt (v 1.14). For total and small RNA-seq analyses, reads were aligned to the human genome (UCSC GRCh38) using HiSat2 (v 2.1.0; (32)) or Bowtie2 (V 2.3.2; (33)), respectively. FeatureCounts (v 1.5.3; (34)) was used for summarizing gene-mapped reads. Ensembl (GRCh38.89; (35)) or miRBase (v 21; (36)) was used for annotations. Differential gene expression (DE) was determined by the R package edgeR (v 3.18.1; (37)) using TMM normalization.

### MicroRNA-target predictions

MiRWALK 2.0 (38) was used for the analysis of transcript-specific miRNA-targeting (Supplementary Table ST3). The positions of MTSs in the 3′UTR of mRNAs were derived from TargetScan.

### CLIP data analysis and CLIP scores

Peak coordinates from publicly available CLIP data (15–17), obtained from ENCODE, NCBI GEO and CLIPdb, were mapped to cis-elements (5′UTR, CDS and 3′UTR) of all annotated genes (RefSeq hg19). Cis-element specific CLIP scores were calculated as the number of datasets reporting CLIP peaks mapped to the 5′UTR, coding sequence (CDS) or 3′UTR, as previously reported (39). Thus, the CLIP score indicates the conservation of binding of a RNA-binding protein to a cis-element of a specific mRNA. For IGF2BP1, the following number of datasets was considered, resulting in CLIP scores ranging from 0 to 8: 2 PAR-CLIP (HEK293), 2 eCLIP (hESCs), 2 eCLIP (HepG2), 2 eCLIP (K562). For IGF2BP2 (CLIP score: 0–7): 2 eCLIP (hESCs), 2 eCLIP (K562), 2 iCLIP (K562), 1 PAR-CLIP (HEK293). For IGF2BP3 (CLIP score: 0–6): 1 eCLIP (hESCs), 2 eCLIP (HepG2), 2 iCLIP (K562), 1 PAR-CLIP (HEK293).

#### ChIP-seq data analyses

Genomic promoter-binding sites of SRF were derived from five publicly available ChIP-seq data performed in MEFs (20,40) (two studies), Ewing sarcoma-derived cells (41), H1 hESC (human embryonic stem cells) and the human lymphoblastoid cell line GM12878 (42). ChIP-scores were calculated as the number of datasets reporting ChIP-peaks mapped to the promoter region of a specific gene.

#### Kaplan–Meier and gene expression correlation analyses

Hazardous ratios (HRs) for indicated gene panels and tumor cohorts of serous ovarian carcinoma, lung adenocarcinoma (LUAD) and HCC were determined by the Kaplan–Meier (KM) plotter (www.kmplot.com) (43) online tool using best cutoff analyses and the multigene classifier.

#### Gene expression correlations

The correlation of gene expression was determined using the R2 platform (http://hgserver1.amc.nl/cgi-bin/r2/main.cgi) to analyze the indicated TCGA-provided datasets (ovarian serous cystadenocarcinoma, LUAD, liver HCC and skin cutaneous melanoma). Pearson correlation coefficients (*R* values) are summarized in Supplementary Table ST4B.

### Cell culture, transfection and CRISPR/Cas9

Cells were cultured and transfected essentially as described recently (9). SiRNAs are summarized in Supplementary Table ST6. For the depletion of DICER1/DROSHA, cells were retransfected after 3 d and harvested 6 d after the initial transfection, as recently described (6). IGF2BP1 knockout cells were generated using the CRISPR/Cas9 technology and sgRNAs previously described (6). Control clones were generated by transfecting the Cas9 nuclease only. For the deletion of the bulk 3′UTR of SRF (Acc. No.: NM_003131), two CRISPR guide RNAs were used as depicted in Figure 2B. The deletion was validated by PCR amplification of the genomic locus and sequencing. Guide RNAs and PCR primers are summarized in Supplementary Table ST5.

### Spheroid growth, invasion and anoikis resistance

The analyses of 3D spheroid growth, anoikis-resistance and spheroid invasion were performed as previously described (6,9). In brief, for spheroid growth and invasion 1000 cells per well (24 h post-transfection) were seeded in an ultra-low attachment round bottom 96-well plate (Corning 7007) using FBS-containing (10%) DMEM medium. Spheroid growth was monitored for 5 d by light microscopy, and viability was determined by CellTiter-GLO (Promega). Upon spheroid formation (24 h), the invasion matrix (Trevigen; 5 mg/ml) was added to monitor tumor cell infiltration for another 24 h using light microscopy. For anoikis resistance, 1000 cells per well were seeded in an ultra-low attachment flat bottom 96-well plate (Corning 3474) using Dulbecco's Modified Eagle Medium (DMEM) containing 1% fetal bovine serum (FBS). Cell growth was monitored for 5 d, and cell viability was determined as described above.

## RESULTS

### IGF2BP1 promotes SRF expression in cancer cells

**Figure 1.**
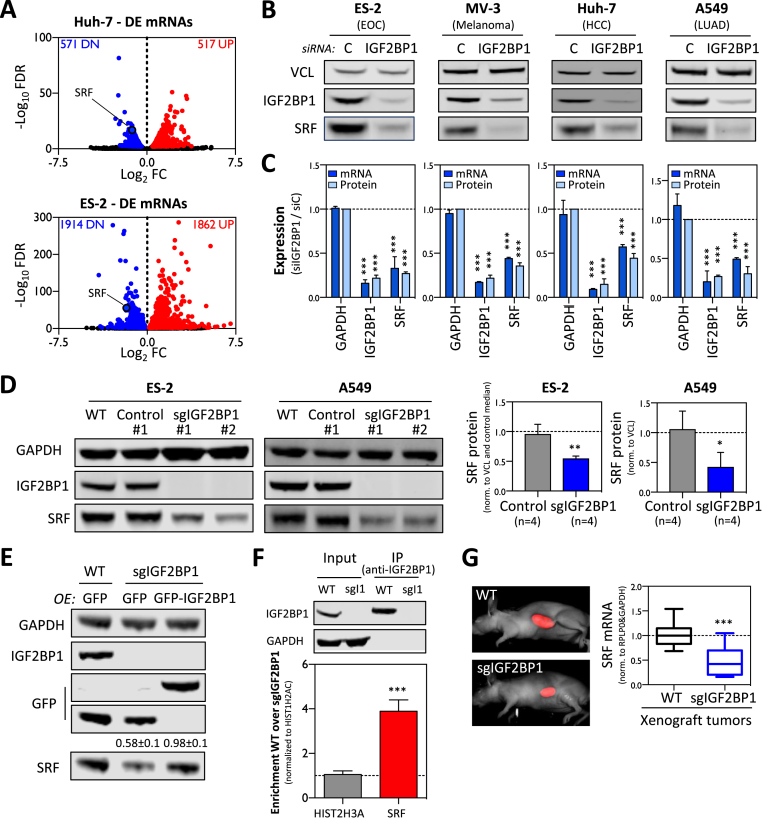
IGF2BP1 promotes SRF expression in cancer cells. (**A**) Volcano plots showing differential gene expression (threshold: FDR ≤ 0.01) determined by RNA-seq in Huh-7 and ES-2 cells upon IGF2BP1 depletion by siRNA pools (72 h). (**B**) Representative western blots demonstrating downregulation of SRF protein upon IGF2BP1 depletion by siRNA pools (72 h) in indicated tumor-derived cell lines. (**C**) Quantification of SRF protein and mRNA abundance upon IGF2BP1 depletion in cancer cells shown in (B). VCL served as the normalization control in three independent western blot analyses. GAPDH served as the negative control in RT-qPCR studies cross-normalized to RPLP0 expression. (**D**) Representative western blot analysis (left panel) demonstrating deletion of IGF2BP1 by CRISPR/Cas9 in two independent cell clones of ES-2 and A549 cells (sgIGF2BP1) compared to parental cells (WT) or Cas9-transfected control clones (C-1). The quantification of SRF protein levels in four control and IGF2BP1-deleted A549 and ES-2 cell clones confirmed that the deletion of IGF2BP1 results in significantly reduced SRF protein expression. GAPDH served as the loading and normalization control. (**E**) Western blotting analyses indicate that the re-expression of wild-type GFP-fused IGF2BP1 restores the expression of SRF protein in IGF2BP1-deleted ES-2 cells. GAPDH served as the loading and normalization control for the quantification of SRF protein levels in three independent studies (indicated above lower panel). (**F**) RIP analysis showing that IGF2BP1 is associated with the SRF mRNA in ES-2 cells. RNA co-purified with IGF2BP1 from parental (WT) or IGF2BP1-KO (sgI1) cells was analyzed by RT-qPCR. HIST1H2AC served as the normalization and HIST2H3A as the negative control. Error bars indicate standard deviation determined in at least three analyses. (**G**) The quantification of SRF mRNA abundance in ES-2 derived *Xenograft* tumors grafted in nude mice by RT-qPCR indicates that IGF2BP1 deletion is associated with reduced SRF expression (right panel). GAPDH and RPLP0 served as normalization controls. Unpublished images of iRFP-labeled tumors (left panel) were derived from recent studies (6). Statistical significance, as indicated by *P*-values, was determined by Student’s *t*-test: (*) *P* < 0.05, (**) *P* < 0.01, (***) *P* < 0.001.

The oncofetal mRNA-binding protein IGF2BP1 is a post-transcriptional enhancer of oncogene expression impairing the miRNA-directed degradation of its target mRNAs (6,9). To identify conserved effector networks of IGF2BP1 in HCC and EOC, IGF2BP1-dependent gene expression was analyzed in HCC-derived Huh-7 and EOC-derived ES-2 cells. To this end, mRNA abundance was monitored by RNA-seq upon the depletion of IGF2BP1 using homolog-specific siRNA pools (Figure 1A and Supplementary Table S1 (6)). The number of differentially expressed genes was higher in ES-2 than in Huh-7 cells. In part, variable effects on gene expression were expected since IGF2BP1 controls target mRNA abundance in a miRNome-dependent manner (6), and miRNA expression (the miRNome) varies between distinct tumor cell lines. Small RNA-seq confirmed a partially distinct miRNA expression in both cell lines, for instance significantly lower abundance of the let-7-5p family but elevated expression of the hepatic miR-122-5p in Huh-7 cells (Supplementary Table ST2 and Supplementary Figure S1A,B). In support of a miRNome-dependent regulation, the absolute number of let-7 target mRNAs (predicted by at least two out of four databases using MirWalk (38)) downregulated by IGF2BP1 depletion was higher in ES-2 (429) than Huh-7 (158) cells (Supplementary Figure S1C). However, IGF2BP1 interferes with miRNA-directed mRNA degradation mostly independent of primary MTS sequences. The protein preferentially associates upstream of MTSs in the 3′UTR of its target mRNAs and recruits associated transcripts to miRNA-/RISC-free mRNPs (6,9). To analyze this for the here suggested target mRNAs of IGF2BP1, we considered eight CLIP-seq (cross-linking immunoprecipitation high-throughput sequencing) studies. The CLIP score indicating the number of experiments featuring cross-link peaks between IGF2BP1 in the 5′UTR, CDS or 3′UTR of specific mRNAs was introduced to rate CLIP-reported mRNA-binding (39). This allowed the comprehensive assessment of preferred binding regions irrespective of distinct CLIP techniques and cell lines used in individual analyses. The IGF2BP1 3′UTR CLIP score was significantly higher among transcripts downregulated (DN) upon IGF2BP1 depletion in both cell lines (Supplementary Figure S1D). This suggested that mRNAs significantly downregulated in both cell lines are more likely direct target transcripts and thus effectors of IGF2BP1 that are conserved in HCC and EOC. To evaluate if IGF2BP1 depletion affected similar pathways despite the only moderate overlap of transcripts significantly (false discovery rate (FDR) < 0.01) deregulated in both cell lines, gene set enrichment analyses were performed. These revealed a striking overlap of hallmark pathways affected by the knockdown of IGF2BP1 in both cell lines (Supplementary Figure S1E and Supplementary Table ST1B). In conclusion, these findings suggested that although IGF2BP1 may have variable regulatory ‘potency’ on specific mRNAs in distinct cancer cells, it serves conserved functions and controls similar pathways at varying extend or significance in these cells.

Among the various transcripts downregulated in both cancer cell lines (242) was the SRF (serum response factor) mRNA encoding a transcriptional regulator modulating both proliferative and migratory/invasive tumor cell properties in a conserved manner, as previously demonstrated for IGF2BP1 (6,7). The expected similarities and conservation of SRF’s and IGF2BP1’s roles in modulating tumor cell properties suggested that the post-transcriptional regulator IGF2BP1 synergizes with the transcriptional regulator SRF in promoting an ‘aggressive’ tumor cell phenotype. Therefore, the conservation of IGF2BP1-dependent regulation of SRF expression was analyzed in a panel of four tumor cell lines derived from distinct primary cancers. These studies revealed that SRF mRNA and protein abundance was significantly decreased in all cell lines upon IGF2BP1 knockdown (Figure 1B and C). This was confirmed in additional tumor-derived cell lines (data not shown) demonstrating that the IGF2BP1-dependent upregulation of SRF expression is highly conserved in cancer cells. To validate regulation of SRF expression by IGF2BP1, the latter was deleted in ES-2 (EOC) and A549 (LUAD) cells using CRISPR/Cas9 technology. In both cell lines, the knockout of IGF2BP1 was associated with decreased SRF expression in four independent cell clones of each cell line (Figure 1D). To exclude bias by off-target effects and validate that IGF2BP1 promotes SRF expression in a RNA-binding-dependent manner, SRF protein and mRNA abundance were monitored by knockdown recovery studies (Supplementary Figure S2A). For this, ES-2 cells were depleted for IGF2BP1 using siRNAs directed against the human IGF2BP1-encoding mRNA resulting in severely reduced SRF expression. SRF protein and mRNA levels were substantially increased by the re-expression of GFP-fused wild-type chicken Igf2bp1 (chI1; also termed ZBP1). On the contrary, SRF abundance remained reduced when re-expressing a GFP-tagged, RNA-binding deficient mutant of chicken Igf2bp1 (chI1 mut) (44), as observed in cells expressing GFP alone (control). Furthermore, SRF expression was restored in IGF2BP1-deleted ES-2 cells by the re-expression of human GFP-tagged IGF2BP1, whereas SRF abundance remained reduced in IGF2BP1-deleted cells transduced with GFP alone (Figure 1E). These findings excluded off-target effects of the used siRNA pool as well as sgRNAs used for IGF2BP1 deletion and suggested that IGF2BP1 controls SRF expression in a RNA-binding dependent manner. To elucidate if IGF2BP1 associates with the SRF mRNA in ES-2 cells, binding was analyzed by RIP. In contrast to the control transcript HIST2H3A, the SRF mRNA was significantly enriched with IGF2BP1 from parental, wild-type (WT) but not IGF2BP1-deleted (sgI1) ES-2 cells (Figure 1F). Finally, downregulated SRF synthesis upon IGF2BP1 deletion was also observed in ES-2-derived *Xenograft* tumors in nude mice (Figure 1G; tumor samples were obtained from (6)). This suggested that the IGF2BP1-dependent enhancement of SRF expression is associated with the recently reported role of IGF2BP1 in promoting tumor growth and metastasis (6).

### IGF2BP1 promotes SRF expression in a 3′UTR and m^6^A-dependent manner

**Figure 2.**
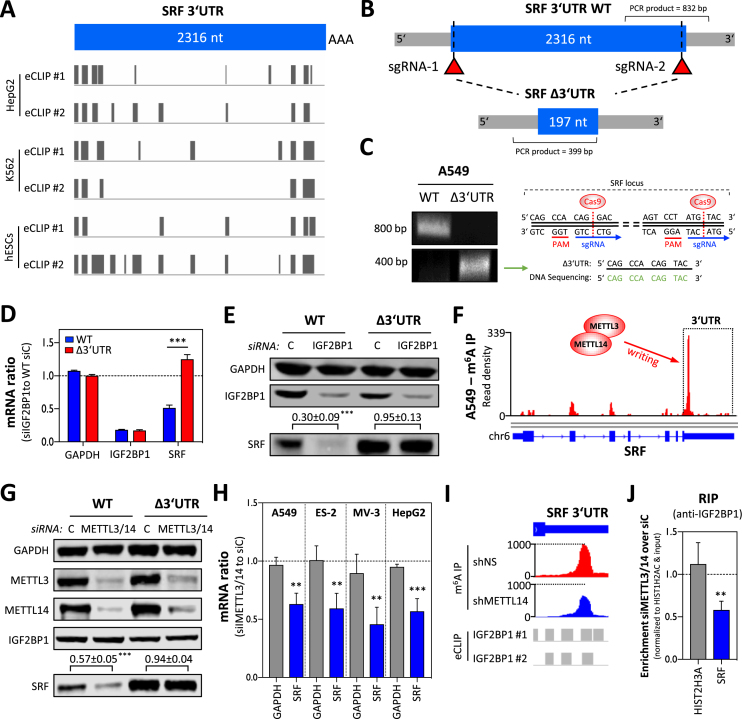
IGF2BP1 promotes SRF expression in a 3′UTR and m^6^A-dependent manner. (**A**) Schematic depicting the position of IGF2BP1-CLIP sites reported in the SRF 3′UTR by six experiments performed in three indicated cell lines. (**B**) Schematic showing the SRF-3′UTR deletion strategy by CRISPR/Cas9. The relative position of sgRNAs and PCR primers for validating deletion of the SRF 3′UTR are indicated. (**C**) Representative semi-quantitative PCR analysis (left panel) of parental (WT) and SRF-3′UTR-deleted (Δ3′UTR) A549 cells. The successful deletion was further validated by DNA sequencing (right panel) of PCR products (Δ3′UTR) spanning the expected cleavage sites indicated in the schematic. (**D**) RT-qPCR analysis of indicated mRNAs in parental and SRF-Δ3′UTR A549 cells upon IGF2BP1 depletion (72 h). RPLP0 served as the normalization and GAPDH as the negative control. (**E**) Representative western blot analysis of indicated proteins in cells treated as described in (D). GAPDH served as the loading and normalization control for the quantification (*n* = 3) of SRF protein levels upon IGF2BP1 depletion (relative to controls), as depicted above lower panel. (**F**) m^6^A -RIP-seq data showing m^6^A-modification of the SRF mRNA in A549 cells. Sequencing data were obtained from MeT-DB V2.0 (45). (**G**) Representative western blot analysis of indicated proteins upon METTL3/14 depletion in parental (WT) and SRF-Δ3′UTR A549 cells. Note that IGF2BP1 expression is unaffected by METTL3/14 depletion, whereas SRF protein abundance is decreased only in parental A549 cells. GAPDH served as the loading and normalization control for the quantification (*n* = 3) of SRF protein levels (relative to controls), as indicated in the lower panel. (**H**) The depletion of METTL3/14 by siRNA pools impairs SRF mRNA abundance in indicated cell lines. GAPDH served as the negative control in RT-qPCR studies cross-normalized to RPLP0 expression. (**I**) Altered m^6^A-modification of the SRF 3′UTR was determined upon METTL14 depletion in HepG2 cells by m^6^A-RIP-seq. Sequencing data were deposited according to (14). Note that the m^6^A-modified region partially overlaps with IGF2BP1-CLIP sites determined in HepG2 cells. (**J**) IGF2BP1-RIP analyses showing reduced association of the SRF mRNA with IGF2BP1 in METTL3/14-depleted A549 cells. RNA co-purified with IGF2BP1 from cells transfected with control siRNAs (siC) or METTL3/14-depleted cells was analyzed by RT-qPCR. HIST1H2AC served as the normalization and HIST2H3A as the negative control. Error bars indicate standard deviation determined in at least three analyses. Statistical significance was determined by Student’s *t*-test: (**) *P* < 0.01, (***) *P* < 0.001.

Consistent with IGF2BP1-CLIP studies in distinct cell lines (15–17), we recently demonstrated that IGF2BP1 impairs the miRNA-dependent downregulation of target mRNAs mainly by associating with the 3′UTR of target transcripts (6). To test if this is also observed for the SRF mRNA, three IGF2BP1-CLIP studies performed in HCC-derived HepG2, leukemia-derived K562 or hESCs were considered (16,17). In all three cell models, IGF2BP1-CLIP hits were identified in the 3′UTR of the SRF mRNA suggesting conserved regulation via this cis-element (Figure 2A). The conservation of IGF2BP1-dependent regulation was further supported by substantially decreased SRF mRNA and protein levels upon the depletion of IGF2BP1 in HepG2 cells (Supplementary Figure S3A and B). Aiming to validate regulation via the ‘endogenous’ 3′UTR, the vast majority of this cis-element in the *SRF* locus was deleted in A549 cells by directing Cas9 nuclease to the proximal and distal (located upstream of the polyadenylation signal) ends of the 3′UTR using two sgRNAs (Figure 2B). The biallelic deletion of the bulk 3′UTR (SRF-Δ3′UTR) was confirmed by PCR (Figure 2C). Compared to parental (WT) cells, SRF mRNA and protein abundance was substantially increased in SRF-Δ3′UTR cells suggesting that the 3′UTR essentially accounts for limiting SRF expression (Figure 2D and E; Supplementary Figure S3C). To test if the 3′UTR-dependent downregulation of SRF synthesis is IGF2BP1-dependent, IGF2BP1 was depleted in parental and SRF-Δ3′UTR cells (Figure 2D and E). Whereas SRF mRNA and protein abundance remained largely unchanged in SRF-Δ3′UTR cells, SRF expression was significantly reduced in parental cells upon IGF2BP1 depletion. This indicated that the IGF2BP1-dependent regulation of SRF expression is strictly 3′UTR-dependent.

In recent studies, IGF2BPs were reported to enhance the expression of MYC and other target transcripts in a m^6^A-dependent manner (14). The m^6^A-modification of the MYC mRNA promotes the association of IGF2BP1 resulting in reduced decay of the MYC transcript and consequently enhanced the expression of this oncogene in cancer cells. The analysis of publicly available m^6^A-RIP-seq data in A549 cells in the MeT-DB V2.0 database (45) indicated strong modification in the 3′UTR of the SRF mRNA (Figure 2F). To test if SRF expression is controlled in a m^6^A- and 3′UTR-dependent manner, the methyltransferases METTL3 and 14 were co-depleted in parental and SRF-Δ3′UTR A549 cells. The knockdown of METTL3/14 resulted in a significant downregulation of SRF protein in parental cells (Figure 2G). In contrast, SRF protein levels remained unaltered in SRF-Δ3′UTR A549 cells upon METTL3/14 depletion. This suggested that SRF expression is controlled via m^6^A-modification in the 3′UTR of the SRF mRNA. If the m^6^A-dependent regulation of SRF expression is conserved, it was investigated by monitoring SRF mRNA and protein abundance upon METTL3/14 depletion in four cancer-derived cells (Figure 2H and Supplementary Figure S3D). In all analyzed cell lines, SRF mRNA and protein abundance was significantly reduced upon the co-depletion of METTL3/14. As observed in A549 cells, m^6^A-RIP-seq analyses in HepG2 cells confirmed that the SRF mRNA is modified in the 3′UTR and that modification is reduced by the depletion of METTL14 (Figure 2I; m^6^A-RIP-seq data were obtained from Huang *et al.* (14)). Notably, m^6^A-modified nucleotides largely overlap with reported IGF2BP1-CLIP sites in HepG2 cells suggesting that the IGF2BP1-dependent regulation of SRF expression is m^6^A-dependent (Figure 2I and Supplementary Figure S3E). If the depletion of METTL3/14 impairs the association of IGF2BP1 with the SRF mRNA, as reported for the MYC mRNA (14), it was analyzed by RIP. Compared to cells transfected with control siRNAs, the co-depletion significantly reduced the association of the SRF mRNA with IGF2BP1 (Figure 2J and Supplementary Figure S3F). These findings supported the view that the m^6^A-modification of the SRF mRNA promotes its association with IGF2BP1, as previously reported for the MYC mRNA (14). However, IGF2BP1 also binds mRNAs independent of m^6^A, e.g. (14,44). Therefore, we hypothesized that reduced m^6^A-modification is partially compensated by increasing IGF2BP1 abundance. If the elevated abundance of IGF2BP1 restores SRF expression when METTL3 is depleted, it was analyzed in ES-2 cells stably overexpressing GFP (control), wild-type (I1) or RNA-binding deficient (I1mut) IGF2BP1. Upon METTL3 knockdown, SRF protein abundance was substantially enhanced in cells overexpressing GFP-IGF2BP1 when compared to I1mut- or GFP-expressing controls (Supplementary Figure S3G). These findings indicate that SRF expression is enhanced by IGF2BP1 in a conserved, 3′UTR- and m^6^A-dependent manner.

### IGF2BP1 impairs the miRNA-dependent downregulation of SRF expression

**Figure 3.**
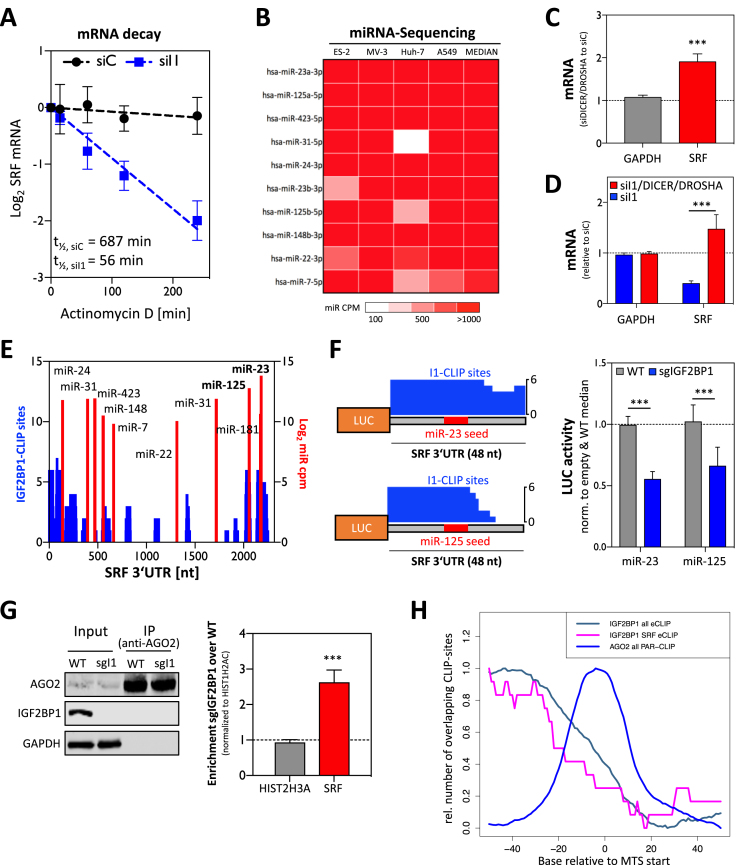
IGF2BP1 promotes SRF expression in a miRNA-dependent manner. (**A**) The decay of the SRF mRNA was monitored by RT-qPCR in ES-2 cells treated with actinomycin D for the indicated time upon transfection of control (siC) or IGF2BP1-directed siRNA pools. The reduction of the SRF mRNA half-life by IGF2BP1 knockdown is indicated in the graph. (**B**) Heatmap indicating the expression of the 10 most abundant and ‘conserved’ miRNAs, predicted to target the SRF 3′UTR, in the analyzed tumor-derived cell lines. MiRNAs are sorted by median expression (CPM, count per million) and color codes of mRNA abundance are shown in the lower panel. (**C**) RT-qPCR analysis demonstrating the upregulation (relative to controls, siC-transfected) of the SRF mRNA upon DICER/DROSHA depletion in ES-2 cells. GAPDH served as the negative and RPLP0 as the normalization control. (**D**) RT-qPCR analysis of SRF mRNA levels upon IGF2BP1 or IGF2BP1/DICER/DROSHA depletion relative to controls (siC-transfected). RPLP0 served as the normalization control and GAPDH as the negative control. (**E**) The number of CLIP studies showing overlapping IGF2BP1-CLIP sites (CLIP score at nucleotide resolution) in the SRF 3′UTR (blue), and the position (*x*-axis) of miRNA targeting sites (red) are shown for the SRF 3′UTR. MiRNA abundance (right axis, red) is indicated as log_2_ CPM for 10 ‘SRF-targeting’ miRNAs showing conserved expression in the cancer cells analyzed. (**F**) Schematic (left panel) showing luciferase reporter constructs comprising indicated regions of the SRF 3′UTR including predicted MTSs for miR-23a-3p and miR-125a-5p. Note that miR-seeds (red) overlap with IGF2BP1-binding sites (blue) suggested by eCLIP analyses (CLIP score at nucleotide resolution is indicated). Luciferase reporter analysis (right panel) demonstrating reduced activity of indicated reporters in IGF2BP1-deleted (sgIGF2BP1, blue) compared to parental ES-2 cells (grey). (**G**) RIP analysis showing the enhanced association of the SRF mRNA with AGO2 in IGF2BP1-deleted ES-2 cells. Immunoprecipitation was analyzed by western blotting (left panel). RNA co-purified with AGO2 from WT or IGF2BP1-deleted (sgI1) ES-2 cells was analyzed by RT-qPCR (right panel). HIST1H2AC served as the normalization and HIST2H3A as the negative control. (**H**) The relative number of overlapping CLIP sites determined for IGF2BP1 and AGO2 in the proximity of MTSs, as recently reported (6), is shown relative to the start of MTSs predicted by TargetScan for human mRNAs (hg19; IGF2BP1 all eCLIP) or the SRF 3′UTR (IGF2BP1 SRF eCLIP). Error bars indicate standard deviation determined in at least three analyses. Statistical significance was determined by Student's *t*-test: (***) *P* < 0.001.

Recent studies indicate that IGF2BPs control mRNA turnover largely by modulating the miRNA-dependent regulation of their target transcripts. Whereas IGF2BP3 was shown to promote or impair the miRNA-directed downregulation of target mRNAs (46), IGF2BP1 and 2 interfere with the miRNA-directed inhibition of effector expression (3,6,9). Although CLIP-hits in the 3′UTR of the SRF mRNA were reported for all three IGF2BPs, only the depletion of IGF2BP1 interfered with the expression of SRF in cancer cells (Supplementary Figure S4A and B). In agreement, IGF2BP1 expression showed the most significant and conserved association with elevated SRF expression in ovarian, skin, liver and lung cancer, as determined by Pearson correlation of RNA-sequencing data available via the TCGA (Supplementary Figure S4C). In view of recent reports, these findings suggested that IGF2BP1 promotes SRF expression in cancer by impairing the miRNA-dependent decay of the SRF mRNA. Consistently, the depletion of IGF2BP1 led to significantly enhanced decay of the SRF mRNA in ES-2 cells (Figure 3A). The analysis of miRNA expression by small RNA-seq revealed that miRNAs, predicted to target the SRF-3′UTR (3 of 4 analyzed databases; Supplementary Table ST3), showed conserved expression (median CPM (counts per million mapped reads)) in the four tumor cell lines for which the IGF2BP1-dependent control of SRF expression was demonstrated (Figure 3B, only the 10 most abundant miRNAs are shown; Supplementary Figure S4D and Supplementary Table ST2). Among these were miRNAs or miR families like miR-22-3p, 125-5p or miR-181-5p that were previously reported to downregulate SRF expression in cancer, smooth muscle and/or endothelial cells (47–50). If SRF expression is controlled by miRNAs in ES-2 cells, it was investigated by depleting DICER and DROSHA. This depletion resulted in a significant downregulation of bulk miRNA abundance, as recently shown and indicated here for miR-22 by northern blotting (Supplementary Figure S4E (6)). The decrease in miRNA levels by DICER/DROSHA knockdown was associated with a severe upregulation of SRF mRNA abundance (Figure 3C). Moreover, it abolished the downregulation of SRF mRNA levels observed upon the depletion of IGF2BP1 indicating that the protein stabilized the SRF mRNA by impairing its miRNA-directed downregulation (Figure 3D). As observed for other miRNA-controlled target mRNAs of IGF2BP1, for instance SIRT1 (6), not all predicted MTSs overlapped with reported IGF2BP1-binding sites in the SRF 3′UTR (Figure 3E). If IGF2BP1 modulates regulation by the two most abundant miRNAs predicted to target the SRF 3′UTR (miR-23a-3p and miR-125a-5p) was analyzed by luciferase reporters. These comprised 48-nt long fragments of the SRF 3′UTR including the predicted MTSs (Figure 3F, left panel). In ES-2 cells deleted for IGF2BP1, reporter activities were significantly decreased compared to parental cells (Figure 3F, right panel). This suggested that IGF2BP1 impaired miRNA-directed downregulation by associating with the respective SRF-derived elements as reported by CLIP analyses. To test if IGF2BP1 interferes with RISC-association of the SRF mRNA in cells, as previously proposed for other target mRNAs (39), AGO2-RIP studies were performed in parental and IGF2BP1-deleted ES-2 cells. These studies showed that the deletion of IGF2BP1 significantly promotes the association of the SRF mRNA with AGO2 (Figure 3G). Finally, the inspection of IGF2BP1-CLIP sites in the vicinity of MTSs in the SRF 3′UTR confirmed the preference of IGF2BP1-binding upstream of MTSs, as previously reported by transcriptome-wide analyses (Figure 3H (6)). In conclusion, these findings indicate that IGF2BP1 impairs the miRNA-directed downregulation of SRF expression.

Previous studies indicate that IGF2BP1 associates with ELAVL1 (HuR), as well as other RBPs in cytoplasmic mRNPs and controls target mRNA fate, e.g. of the MYC mRNA, in concert with these co-factors (51). ELAVL1 is a key regulator of mRNA turnover and translation promoting or impairing miRNA-directed regulation of its target mRNAs (52). Accordingly, it was tempting to speculate that both proteins cooperate or antagonize each other in the miRNA-dependent regulation of SRF expression. This was analyzed by depleting IGF2BP1 and ELAVL1 in A549 cells. Whereas SRF protein levels were decreased by IGF2BP1 knockdown, they remained unchanged upon the depletion of ELAVL1 (Supplementary Figure S5A). The analysis of IGF2BP1- and ELAVL1-mRNA binding, as reported by CLIP studies, revealed that although both proteins preferentially associate in the 5′-proximity of MTSs they show substantially distinct binding properties at MTSs and the 3′-proximity of miRNA targeting sites (Supplementary Figure S5B). Although these findings do not exclude that ELAVL1 and IGF2BP1 co-regulate the miRNA-dependent regulation of some mRNAs, they provide strong evidence that the IGF2BP1-dependent regulation of the SRF mRNA is independent of ELAVL1.

### IGF2BP1 promotes SRF-dependent transcription in cancer cells

**Figure 4.**
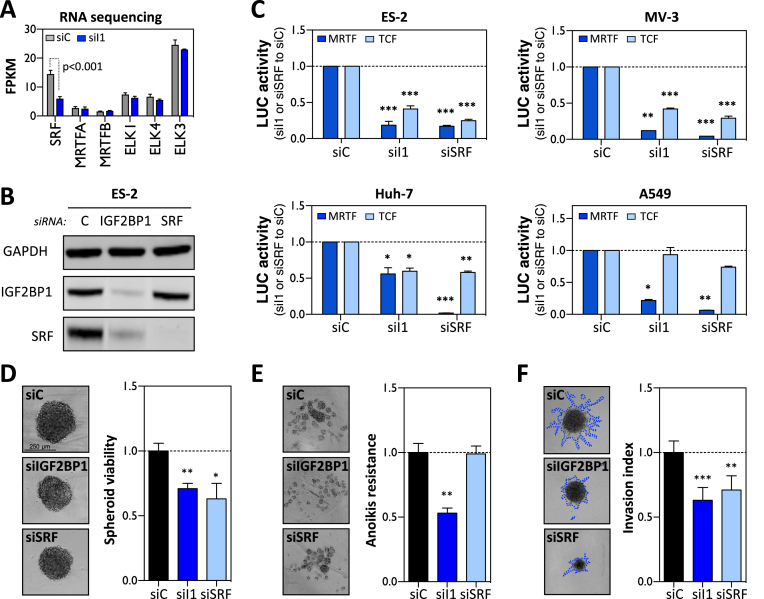
IGF2BP1 promotes SRF-dependent transcription. (**A**) The abundance of indicated mRNAs was determined by RNA-seq (shown as FPKM; fragments per kilobase per million mapped reads) in ES-2 cells transfected with control (siC) or IGF2BP1-directed (siI1) siRNAs. (**B**) Representative western blot analysis of indicated proteins in ES-2 cells transfected with control (siC), IGF2BP1- (siI1) or SRF-directed (siSRF) siRNA pools for 72 h. (**C**) Luciferase reporter assays using MRTF-dependent (dark blue) or TCF-dependent (light blue) promoter constructs in the indicated cell lines upon IGF2BP1- or SRF-depletion by siRNA pools. Reporter activity was determined relative to cells transfected with control (siC) siRNAs, 48 h post-transfection. (**D**) The viability of ES-2 derived spheroids cultured at 10% FBS in concave ultra-low attachment plates was determined by Cell-titer GLO (Promega) 72 h post-transfection with indicated siRNA pools. Cells transfected with control siRNA (siC) served as control and the median viability was set to one. (**E**) Anoikis-resistance of ES-2 cells was determined relative to controls (median set to one) by Cell-titer GLO 72 h post-transfection with indicated siRNA pools. Cells were cultured in planar ultra-low attachment plates at 1% FBS. (**F**) The invasive potential of ES-2 spheroids in 3D matrigel matrix was analyzed 72 h post-transfection of indicated siRNA pools. The relative invasion index (median of controls set to one) was determined by the perimeters of the invasive front (traced by blue dashed line) normalized to spheroid body perimeter. Representative images of cell spheroids are shown in left panels (D–F). Error bars indicate standard deviation determined in at least three analyses. Statistical significance was determined by Student’s *t*-test: (*) *P* < 0.05, (**) *P* < 0.01, (***) *P* < 0.001.

SRF modulates gene expression in concert with two groups of signal-regulated co-factors, TCFs (ELK1, 3 and 4) and MRTFs (MRTFA and MRTFB). In concert with these co-regulators and their upstream signaling cascades, SRF-dependent transcriptional control modulates cell proliferation, contractility and pro-invasive behavior (40). RNA-sequencing indicated that the depletion of IGF2BP1 in ES-2 cells only impaired the expression of SRF whereas the abundance of co-factor encoding mRNAs remained unchanged (Figure 4A). This suggested that IGF2BP1 depletion interferes with SRF/TCF- as well as SRF/MRTF-dependent transcriptional regulation in cancer cells mainly by reducing cellular SRF abundance. This was analyzed by monitoring the activity of SRF/TCF- and SRF/MRTF-dependent luciferase reports in cancer cells upon the depletion of IGF2BP1 or SRF (Figure 4B and C). Notably, IGF2BP1 expression remained unchanged upon SRF depletion. The activity of both reporters was substantially diminished by the depletion of either IGF2BP1 or SRF in all cancer cells analyzed. The only exception was observed in A549 cells in which SRF/TCF-dependent reporters were barely affected by IGF2BP1 or SRF depletion for unknown reasons. In Huh-7 cells, the depletion of IGF2BP1 showed only moderate effects on the activity of MRTF-reporters when compared to the knockdown of SRF. This could be a result of the constitutively high Rho-dependent activation of MRTFs due to DLC-1 deficiency in Huh-7 cells (53). Despite the obvious, cell type-dependent and variable extent of IGF2BP1/SRF-directed regulation, the presented findings suggested that IGF2BP1 and SRF exhibit similar effects on promoting a pro-proliferative and pro-invasive gene expression signature in tumor cells. This regulation was likely to mainly rely on the IGF2BP1-dependent upregulation of SRF expression and the consequent cell type-dependent enhancement of SRF/MRTF- as well as SRF/TCF-controlled transcription. To test if IGF2BP1 and SRF modulate tumor cell viability, spheroid growth was monitored upon their depletion (Figure 4D). The knockdown of both factors substantially decreased the growth of ES-2 derived spheroids when cultured in the presence of 10% FBS indicating that both proteins are essential for tumor cell growth or proliferation (Figure 4D). How IGF2BP1 or SRF influence tumor cell viability at low adhesion and mitogen stimulation (FBS, 1%) was analyzed by anoikis resistance assays upon depleting both factors in ES-2 cells (Figure 4E). Whereas IGF2BP1 knockdown impaired cell viability as previously reported (6), anoikis resistance remained largely unaffected by the depletion of SRF. This revealed that IGF2BP1 also serves SRF-independent roles in cancer cells and thus supports the notion that IGF2BP1 promotes an ‘aggressive’ tumor cell phenotype via pleiotropic effectors (6). SRF-dependent transcriptional regulation is a key modulator of cytoskeletal dynamics and was shown to promote tumor cell invasion and experimental metastasis (26), as recently shown for IGF2BP1 in EOC-derived cells (6). In agreement, the depletion of both factors substantially interfered with spheroid invasion in ES-2 cells (Figure 4F). In summary, these findings demonstrate that IGF2BP1 promotes SRF-dependent transcriptional regulation and that both factors likely synergize in promoting an ‘aggressive’ tumor cell phenotype.

### IGF2BP1 promotes SRF-dependent transcription at the post-transcriptional level in cancer

**Figure 5.**
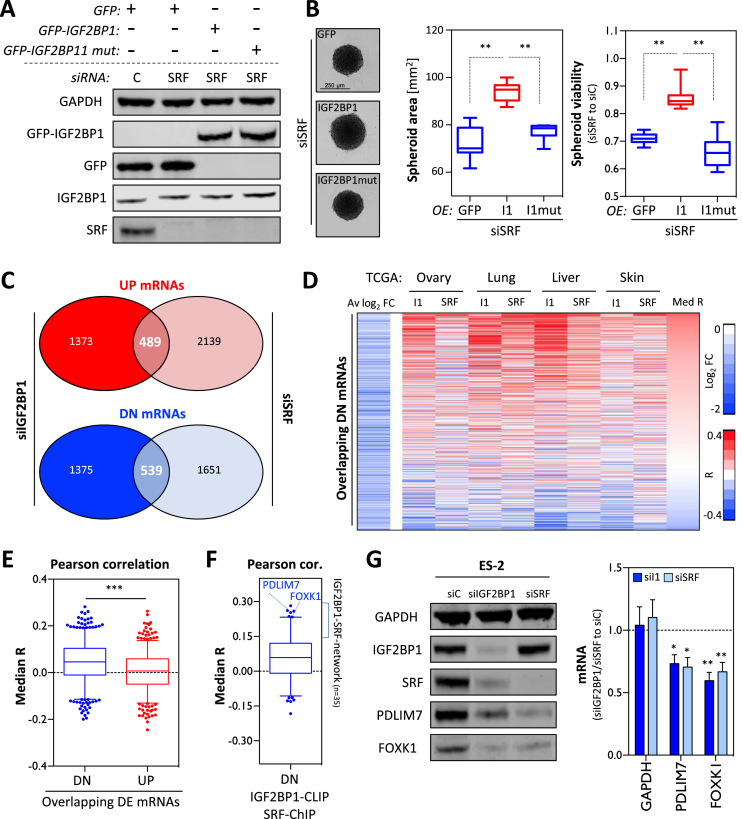
IGF2BP1 and SRF synergize in promoting gene expression in cancer. (**A**) Representative western blot analysis of control (siC) and SRF-depletion (72 h) in ES-2 cells expressing GFP, GFP-IGF2BP1 (GFP-I1) or RNA-binding deficient IGF2BP1 (GFP-I1 mut). (**B**) The maximal area (middle panel) and viability of ES-2 derived spheroids (right panel) transfected as indicated in (A) were derived by inspecting light microscopy images and Cell titer GLO assays (as in Figure 4D), respectively. Representative images are shown in the left panel. Cells transfected with control siRNA (siC) served as control and the median viability was set to one. (**C**) The overlap of mRNAs significantly (FDR ≤ 0.01) up- (UP, red) or downregulated (DN, blue) in ES-2 cells upon the depletion of IGF2BP1 and SRF is shown by Venn diagrams. Numbers indicate transcripts with significantly deregulated expression upon the depletion of IGF2BP1 and/or SRF. (**D**) The expression of genes downregulated by SRF and IGF2BP1 depletion in ES-2 cells was tested by Pearson correlation in indicated cancers using TCGA-derived RNA-seq data. The average log2 fold change (right) of gene expression in ES-2 cells (Av log_2_ FC) upon depletion and correlation coefficients (*R*) determined for IGF2BP1 and SRF in indicated cancers are shown for each downregulated gene by a heatmap. Genes are ranked by the median correlation (Med R) of gene expression (with SRF and IGF2BP1) indicated on the right. Scale bars for the Av log_2_ FC and *R* are shown in the right panel. (**E**) The median *R* values determined as described in (D) are shown by box plots for genes significantly down- (blue) or upregulated (red) upon IGF2BP1 and SRF depletion in ES-2 cells. (**F**) The median *R* values of downregulated genes with a 3′UTR CLIP score ≥ 4 and SRF-ChIP-score ≥ 1 are shown by a box plot. Genes with a median *R* greater 0.15 (I1-SRF-network, *n* = 35), including PDLIM7 and FOXK1, were considered for further analyses. (**G**) Representative western blot (left panel) and RT-qPCR (right panel) analyses of indicated proteins and FOXK1 as well as PDLIM7 mRNAs in ES-2 cells transfected with control (siC), IGF2BP1- or SRF-directed siRNAs (72 h). GAPDH served as the loading control (WB) or the negative control (RT-qPCR). RPLP0 served as the normalization control in RT-qPCR analyses. Error bars indicate standard deviation determined in at least three analyses. Statistical significance was determined by Student’s *t*-test: (*) P < 0.05, (**) *P* < 0.01, (***) *P* < 0.001.

The regulation of SRF-dependent transcription by IGF2BP1 in cancer cells and the partial ‘phenocopy’ observed upon IGF2BP1 and SRF depletion in ES-2 cells suggested that both factors synergize in promoting a pro-proliferative and invasive tumor cell phenotype. In view of reported functions of SRF and IGF2BP1, one plausible molecular mechanism underlying this synergy could be that IGF2BP1 promotes SRF-dependent transcription at the post-transcriptional level by impairing the degradation of SRF-driven transcripts. This implies that elevated IGF2BP1 expression partially restores SRF/IGF2BP1-dependent cell properties in a RNA-binding dependent manner when SRF is reduced. To test this assumption at the phenotypic level, spheroid viability was monitored upon SRF depletion in ES-2 cells expressing GFP, GFP-IGF2BP1 (I1) or a RNA-binding deficient IGF2BP1 (I1mut) mutant (Figure 5A). The analysis of absolute spheroid size (area) and viability (relative to cells transfected with control siRNAs, siC) revealed that wild-type IGF2BP1 significantly enhanced spheroid growth when SRF was depleted (Figure 5B). In contrast, spheroid growth and viability remained unchanged by the overexpression of RNA-binding deficient IGF2BP1 when compared to GFP-expressing controls. This supported the view that elevated IGF2BP1 expression partially compensated for reduced SRF-dependent transcript synthesis by the post-transcriptional stabilization of mRNAs regulated by SRF at the transcriptional level. Aiming to identify transcripts subjected to co-regulation by SRF and IGF2BP1 in ES-2 cells, differential gene expression was monitored by RNA-seq upon the knockdown of SRF (Supplementary Figure S6A and Supplementary Table ST4A). Comparative analysis of differential gene expression upon IGF2BP1 or SRF depletion in ES-2 cells identified a substantial number of genes up- (489) or downregulated (539) by the knockdown of both factors (Figure 5C). To identify conserved candidates for co-regulation by IGF2BP1 and SRF in cancer, the correlation of candidate transcript expression with IGF2BP1 or SRF mRNA abundance in four primary cancers was determined (*R*, Correlation coefficient; Supplementary Table S4B). The median of correlation coefficient was significantly higher for genes downregulated (DN) by the depletion of IGF2BP1 and SRF in ES-2 cells than for upregulated (UP) genes for which the median *R* was slightly above zero (Figure 5E). This suggested that potentially ‘oncogenic’ effectors of SRF/IGF2BP1 are identified by a conserved positive association with SRF/IGF2BP1-expression in cancer and downregulation upon SRF and IGF2BP1 depletion in cancer-derived cells. To identify candidate transcripts enhanced by SRF at the transcriptional level and promoted by IGF2BP1 at the post-transcriptional level, the median *R* of DN-mRNAs with conserved SRF-promoter binding (ChIP-score ≥ 1; Supplementary Table S4C) and IGF2BP1-3′UTR association (CLIP score ≥ 4) was determined (Figure 5F and Supplementary Table S4C). Although SRF-promoter and IGF2BP1-3′UTR association appeared conserved for only 257 of 539 DN-transcripts, the vast majority of these mRNAs showed positively associated expression with SRF and IGF2BP1 in the four cancers analyzed. To validate the oncogenic role of SRF/IGF2BP1-enhanced effector, 35 transcripts with median *R* values above 0.15 determined by correlation analyses, reported SRF-promoter binding and IGF2BP1-3′UTR association were picked for further analyses. Notably, the identified genes included the previously reported IGF2BP1 target mRNA MKI67 (10). Gene annotation enrichment analyses suggested cell proliferation as a major, shared role of the identified genes (Supplementary Table S4C). Two of these 35 mRNAs, PDLIM7 and FOXK1, showing a significant and positive association with both, IGF2BP1 and SRF expression in cancer, as indicated for HCC and EOC (Supplementary Figure S6C), were chosen for validating regulation by IGF2BP1 and SRF.

**Figure 6.**
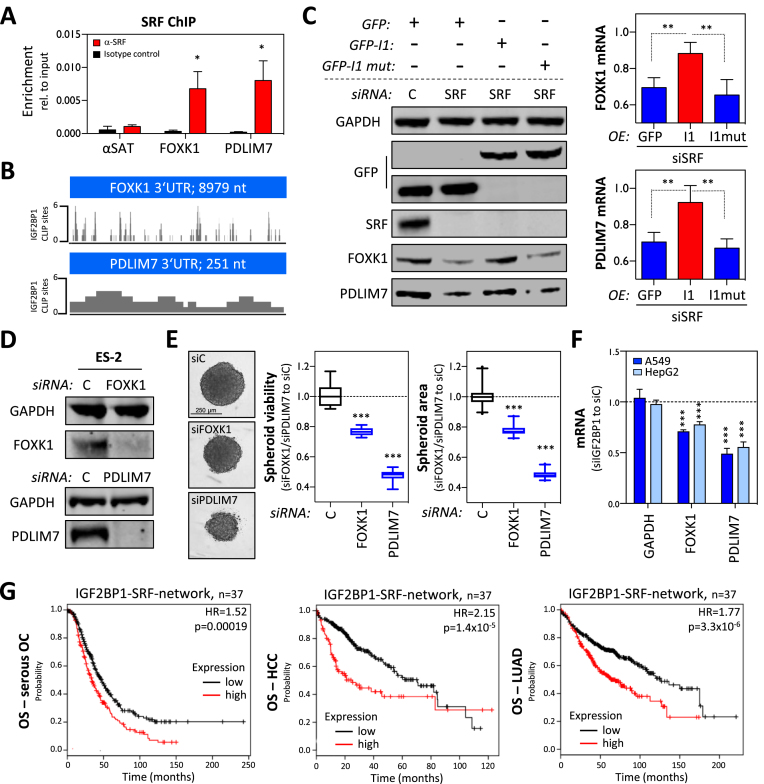
The SRF/IGF2BP1-dependent control of gene expression promotes the expression of ‘oncogenic’ factors. (**A**) SRF-ChIP analysis of FOXK1 and PDLIM7 in ES-2 cells. The enrichment of promoter regions with SRF (red) or isotype control (black) of indicated genes or satellite DNA (SAT; negative control) was determined relative to inputs by qPCR. (**B**) Schematic indicating the position of IGF2BP1-CLIP hits (CLIP score) in the 3′UTR of indicated genes. (**C**) Representative western blot (left panel) and RT-qPCR (right panel) analyses of indicated proteins and mRNAs in ES-2 cells treated as indicated in Figure 5A. GAPDH served as the loading control in WB and the normalization control in RT-qPCR analyses. (**D**) Representative western blot analyses of indicated proteins in ES-2 cells transfected with control (siC), FOXK1- or PDLIM7-directed siRNA pools (72 h). GAPDH served as the loading control. (**E**) The viability and size (area) of ES-2 derived spheroids (right panel) transfected with siRNA pools as indicated in (D) was monitored by Cell-titer GLO 72 h (viability) and light microscopy (size) post-transfection, as described in Figure 4D. Representative images are shown in the left panel. Cells transfected with control siRNA (siC) served as control and the median viability or area was set to one. (**F**) The abundance of indicated mRNAs was determined in A549 or HepG2 cells transfected with control or IGF2BP1-directed siRNA pools (72 h). GAPDH served as the negative and RPLP0 as a normalization control. (**G**) Kaplan–Meier analyses of the 37 I1-SRF-network genes were performed in indicated cancer datasets using the multigene classifier of KM plotter. The OS probability along with HR and *P*-values determined by KM plotter is shown. Error bars indicate standard deviation determined in at least three analyses. Statistical significance was determined by Student’s *t*-test: (*) P < 0.05, (**) *P* < 0.01, (***) *P* < 0.001.

The abundance of PDLIM7 and FOXK1 protein and mRNA was significantly reduced by the knockdown of IGF2BP1 and SRF in ES-2 cells (Figure 5G). SRF-ChIP studies performed in ES-2 cells confirmed the binding of SRF to the promoters of FOXK1 and PDLIM7 providing further evidence that SRF promotes the synthesis of the respective transcripts (Figure 6A). IGF2BP1-CLIP sites reported in HepG2, K562, HEK293 and hESCs suggested that binding of IGF2BP1 to the 3′UTRs of FOXK1 and PDLIM7 is conserved (Figure 6B). To evaluate, if IGF2BP1 restores FOXK1 and PDLIM7 expression when SRF is reduced, as observed in phenotypic analyses (see Figure 5A), ES-2 cells stably expressing GFP, wild-type or RNA-binding deficient IGF2BP1 were transfected with control (siC) or SRF-directed siRNA pools (Figure 6C). The abundance of FOXK1 and PDLIM7 protein and mRNA was significantly increased in cells expressing wild-type IGF2BP1. These findings suggested that IGF2BP1 partially restores the expression of SRF target genes by stabilizing the respective mRNAs. This regulation was associated with a substantial recovery of spheroid growth upon SRF depletion (see Figure 5A and B) suggesting that FOXK1 and PDLIM7 are part of a SRF/IGF2BP1-dependent ‘effector network’ in cancer cells. In support of this, the depletion of FOXK1 and PDLIM7 significantly impaired the growth and viability of ES-2 spheroids, as observed upon the knockdown of IGF2BP1 and SRF (Figure 6D and E). To validate that the regulation of FOXK1 and PDLIM7 by IGF2BP1/SRF is conserved in cancer cells, the abundance of both mRNAs was monitored upon the depletion of IGF2BP1 in two additional cell lines, A549 and HepG2 (Figure 6F). Both transcripts (FOXK1 and PDLIM7) were decreased upon the knockdown of IGF2BP1 suggesting a substantial conservation of SRF/IGF2BP1-dependent regulation of both factors in cancer cells. If the SRF/IGF2BP1-dependent enhancement of FOXK1 and PDLIM7 has prognostic value in cancer, it was evaluated by Kaplan–Meier analyses using KM plotter (43). Next to evaluating the prognostic value of single gene expression, KM plotter also enables gene set studies. In ovarian cancer, elevated expression of the ‘oncogenic gene set’ comprising IGF2BP1, SRF, FOXK1 and PDLIM7 was not significantly (*P* = 0.077) associated with poor overall survival (OS) but showed the expected trend with a HR of 1.22 (Supplementary Figure S7A). Significant prognostic value of the gene set was observed when analyzing progression-free survival (PFS: HR, 1.33; *P* = 0.0059) in ovarian cancer. This was even further pronounced when determining PFS probability only in p53-mutated ovarian cancer where the gene set was significantly associated with a poor prognosis (PFS_p53-mut_: HR, 1.84; *P* = 0.0034). Notably, ES-2 cells were reported to be p53-mutated and were proposed as suitable cell models for studying serous ovarian cancer cell properties (54). Assuming that SRF/IGF2BP1-directed gene expression serves conserved oncogenic roles, we next analyzed the prognostic value of the identified prime candidate gene network comprising 35 genes next to SRF and IGF2BP1 (see Figure 5F and Supplementary Table ST4C). In contrast to the small gene set (IGF2BP1, SRF, FOXK1 and PDLIM7), the enlarged gene set (35 genes plus IGF2BP1 and SRF) was significantly associated with poor OS probability in serous ovarian cancer, HCC as well as LUAD, as supported by HR values ranging from 1.52 to 2.15 (Figure 6G and Supplementary Figure S7B). In conclusion, these findings indicate that the SRF/IGF2BP1-dependent control of gene expression in cancer is largely conserved, promotes the synthesis of factors enhancing tumor cell growth and is associated with unfavorable prognosis in three solid cancers.

## DISCUSSION

Here, we demonstrate that the mRNA-binding protein IGF2BP1 is a conserved post-transcriptional enhancer of SRF-driven transcription in cancer. The protein impairs the miRNA-directed degradation of the SRF mRNA resulting in elevated SRF abundance and transcriptional activity (Figures 1, 3 and 4). Genomic deletion of the bulk 3′UTR of SRF abrogates IGF2BP1-dependent regulation and enhances SRF expression indicating that SRF expression is essentially controlled via its 3′UTR (Figure 2). This observation supports the recently reported major mode of IGF2BP1-directed regulation in cancer cells, the impairment of miRNA-directed downregulation of target mRNAs (6,9). Concomitantly, this observation underpins the physiological relevance of miRNA-directed control of SRF expression reported in cancer cells, endothelial and (smooth) muscle cells, e.g. (47–50).

Recent studies have identified IGF2BPs as novel m^6^A-readers in cancer (14). N^6^-methyladenosine modification in the coding region stability determinant (CRD) of the MYC mRNA enhances the association of IGF2BPs and interferes with the endonuclease-directed decay of the MYC mRNA (11). This enhances the expression of the MYC oncogene in cancer cells, as previously reported in EOC- and HCC-derived cancer cells (10,12). Here, we present the first evidence that SRF expression is enhanced in a IGF2BP1- and m^6^A-dependent manner, as recently reported for MYC. However, in contrast to the latter, m^6^A-/IGF2BP1-dependent regulation is strictly 3′UTR-dependent (Figure 2). This confirms IGF2BP1 as a conserved ‘oncogenic’ m^6^A-reader in cancer and supports the view that IGF2BP1 impairs the miRNA-directed decay of target mRNAs by sequestering transcripts in miRNA-/RISC-free mRNPs (6,9). This mode of regulation is expected to essentially rely on modulating the efficiency, presumably the affinity, of IGF2BP1–mRNA association, as shown here by reduced binding of IGF2BP1 to the SRF mRNA upon METTL3/14 depletion (Figure 2J) and previously demonstrated for the MYC mRNA (14). In an equilibrium, IGF2BP1–mRNA association accordingly relies on the concentration of IGF2BP1 and m^6^A-modified target mRNAs. Consistently, reduced m^6^A-modification is partially compensated when IGF2BP1 abundance is upregulated (Supplementary Figure S3G). Although remaining partially contradictory, m^6^A-modification was proposed to promote oncogenesis in some malignancies including HCC, where METTL3 expression is a significant predictor of poor OS probability (55). Together this suggests that the ‘oncogenic potential’ of IGF2BP1 is enhanced in cancers with upregulated m^6^A-modification in IGF2BP1-target mRNAs.

The conserved regulation of SRF expression by IGF2BP1 essentially relies on the post-transcriptional enhancement of SRF expression that by itself is expected to promote oncogenesis. Recent studies show that the upregulation of SRF enhances pluripotency by interfering with cell identity and induces a metaplasia-like phenotype in the pancreas of transgenic mice (29). The oncogenic role of upregulated SRF expression is further enhanced by the IGF2BP1-dependent sustainment of SRF-target gene expression demonstrated here for PDLIM7 and FOXK1 (Figures 5G and 6C). In agreement with promoting tumor cell vitality (Figure 6E), PDLIM7 was shown to stabilizes MDM2 by interfering with its autoubiquitination resulting in reduced responsiveness toward CDK4/6-inhibition by PD03329921 in cancer cells (56,57). In support of findings presented here (Figure 6E), analyses in EOC-, HCC-derived and other cancer cells indicate that the transcriptional regulator FOXK1 promotes the proliferation and metastatic potential of tumor cells in a conserved manner (58,59). Moreover, FOXK1 synergizes with SRF in controlling the transcription of smooth muscle α-actin and cathepsin-A (60). This suggests that the SRF/IGF2BP1-dependent regulation of gene expression also influences the abundance and/or activity of transcriptional co-regulators of SRF. Whereas FOXK1 is enhanced by SRF/IGF2BP1-dependent regulation, the expression of the two main co-factor groups of SRF, MRTFs and TCFs (18) remained unaffected by IGF2BP1 (Figure 4A). However, IGF2BP1 is a potent post-transcriptional regulator of actin dynamics controlling ACTB protein synthesis as well as MAPK4/MK5/HSP27-dependent regulation of cellular G/F-actin ratios (7,61). Thus, it is tempting to speculate that, next to regulating SRF abundance, IGF2BP1 also modulates the actin-dependent MRTF/SRF (transcriptional) activity, something that needs to be addressed in further detail by follow-up studies. Likewise, IGF2BP1 probably influences the MAPK-modulated activity of SRF/TCF-dependent transcriptional control in cancer cells. IGF2BPs promote the synthesis of growth factors like IGF2 and consistently enhances ERK1/2-activity in liver cancer cells (62,63). At the post-transcriptional level, SRF/IGF2BP1-dependent regulation probably relies on the miRNA-dependent stabilization of SRF-enhanced mRNAs by IGF2BP1. In addition to reports demonstrating miRNA-dependent control of at least FOXK1 (64), the *in silico* prediction of miRNAs targeting PDLIM7 or FOXK1 mRNAs identified various, partially tumor-suppressive miRNAs like members of the let-7-5p, miR-34-5p or miR-181-5p families (data not shown). Taken together, this suggests that IGF2BP1 promotes SRF-dependent transcription in a largely miRNome- and potentially m^6^A-dependent manner in cancer.

The majority of transcripts enhanced by SRF/IGF2BP1 in ES-2 cells show a conserved association with SRF/IGF2BP1-expression in cancer (Figure 5D–F). Their expression is associated with an overall poor survival probability in ovarian, liver and lung cancer supporting the notion that SRF/IGF2BP1-enhanced gene expression is a conserved driver of oncogenesis that promotes both tumor growth and metastasis (6,26). Moreover, SRF/IGF2BP1-driven gene expression may also enhance a stem-like tumor cell phenotype, as supported by the recently reported role of SRF in promoting pluripotency and various studies indicating IGF2BPs to sustain stem-like cell properties (4,29). In conclusion, these findings suggest SRF/IGF2BP1-dependent gene expression as a novel therapeutic hub in cancer treatment. While targeting SRF-dependent transcription likely poses various, broad and undesired side-effects, the targeting of IGF2BP1 may be advantageous. The protein is essentially absent in adult life, and *de novo* synthesis is only observed in cancer. Targeting IGF2BP1 could thus provide a strategy to impair ‘oncogenic’ gene expression including genes enhanced by the co-regulation of SRF/IGF2BP1.

## DATA AVAILABILITY

Total RNA- as well as small RNA-Seq data have been deposited at GEO (GSE116136).

## Supplementary Material

Supplementary Data
