# Supplementary material for: IGF2BP1 promotes SRF-dependent transcription in cancer in a m6A- and miRNA-dependent manner
Source: Nucleic Acids Res. 2018 Oct 29;47(1):375–90. doi: 10.1093/nar/gky1012 (PMC6326824; doi:10.1093/nar/gky1012)
Supplement: Supplementary Data [file gky1012_supplemental_files.zip › Supp_Figures_Rev2.pdf]

Supplementary Figure 1 – Müller et al.

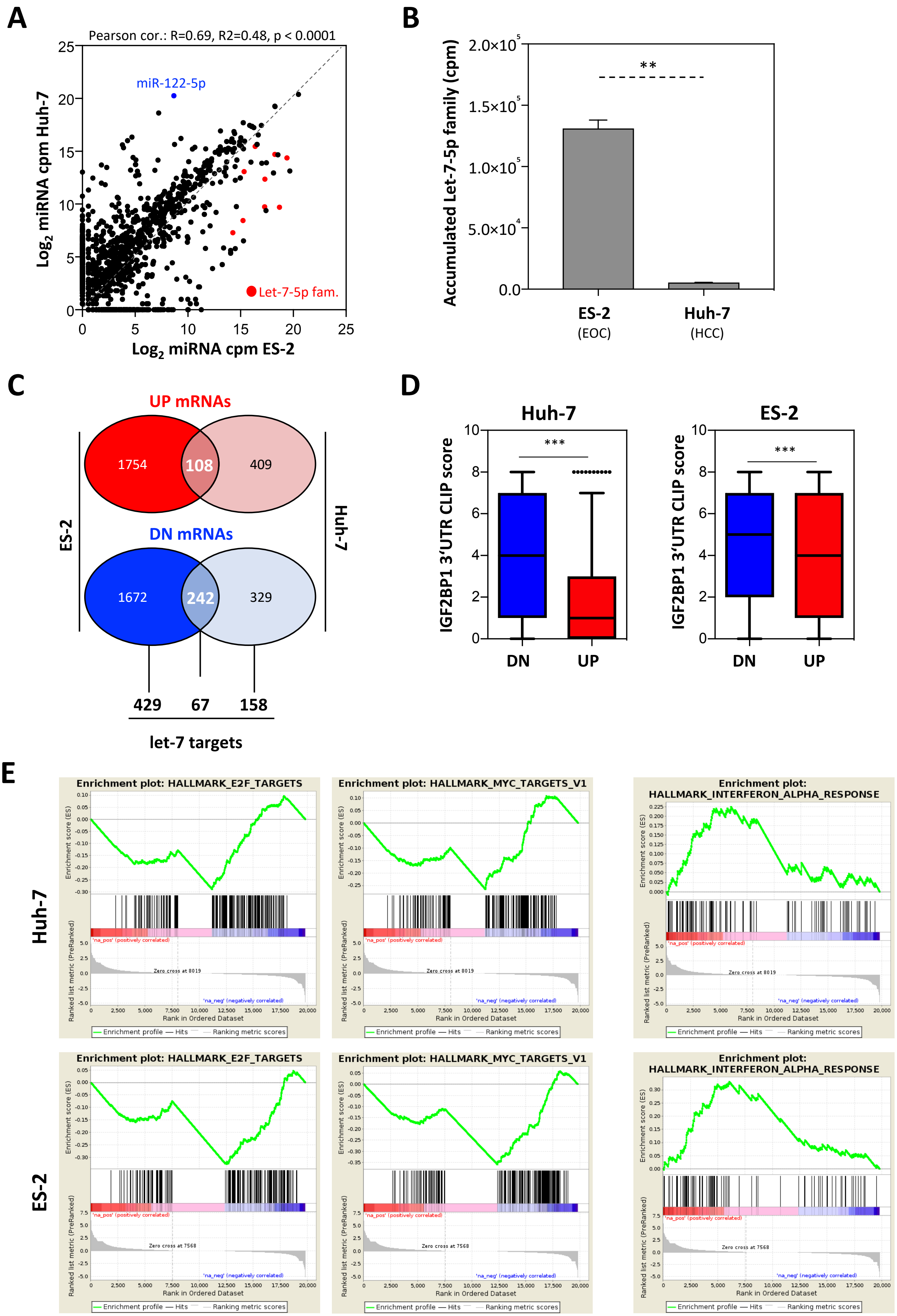

Supplementary Figure 2 – Müller et al.

A

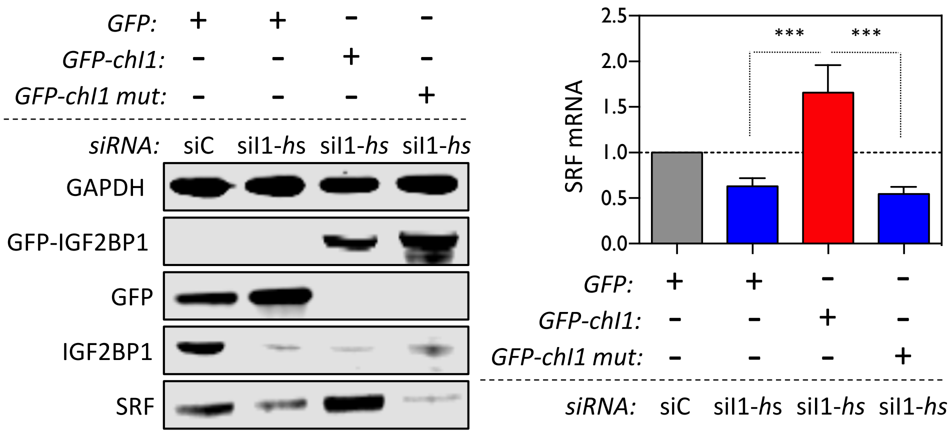

Supplementary Figure 3 – Müller et al.

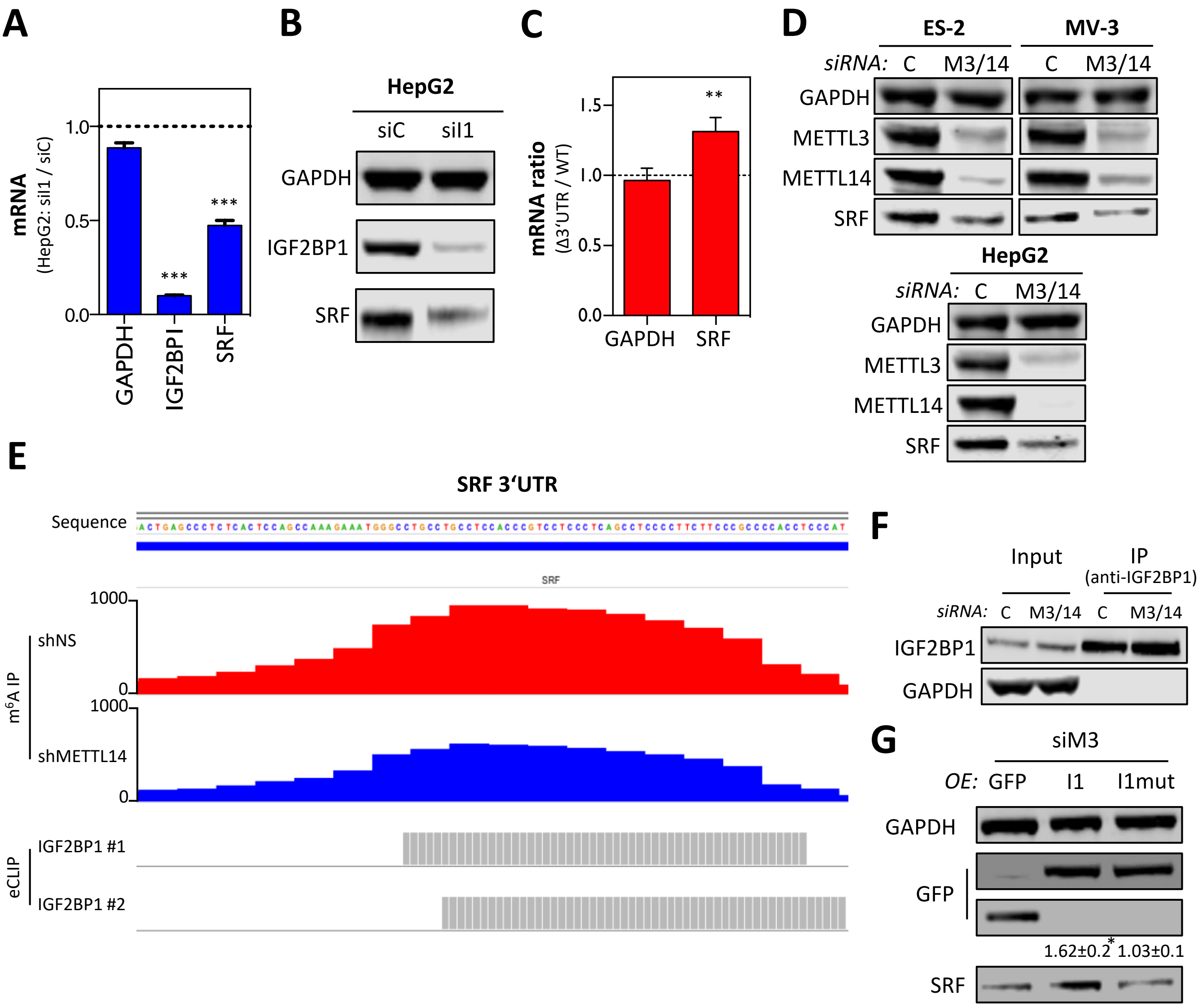

Supplementary Figure 4 – Müller et al.

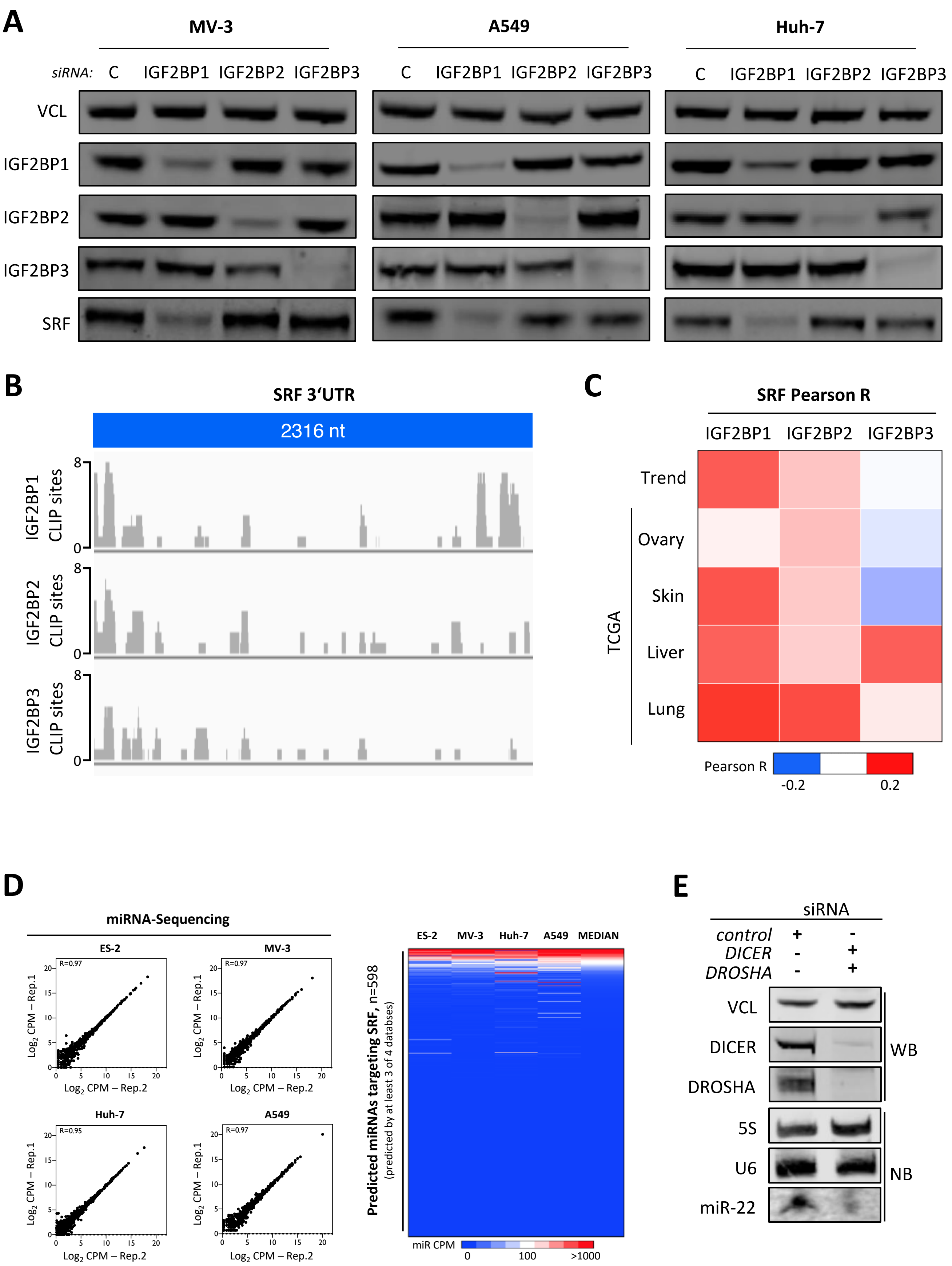

Supplementary Figure 5 – Müller et al.

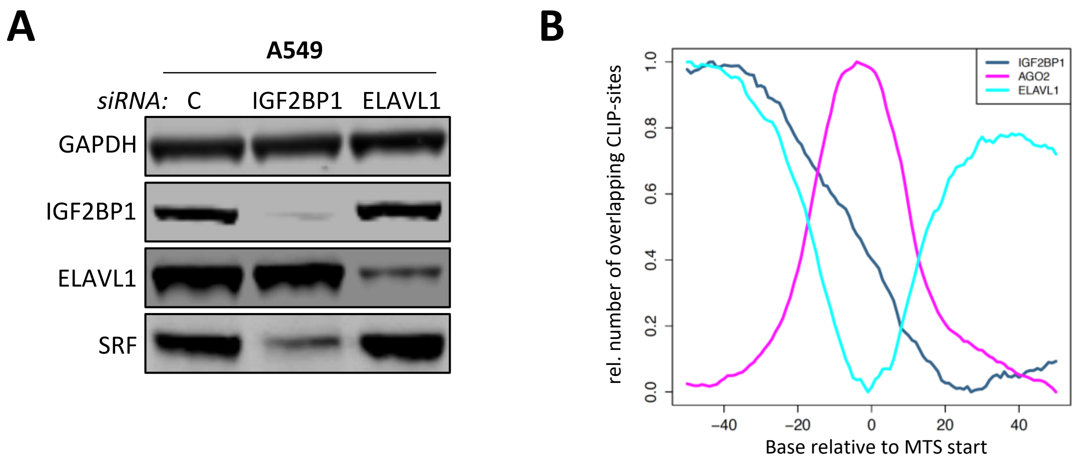

Supplementary Figure 6 – Müller et al.

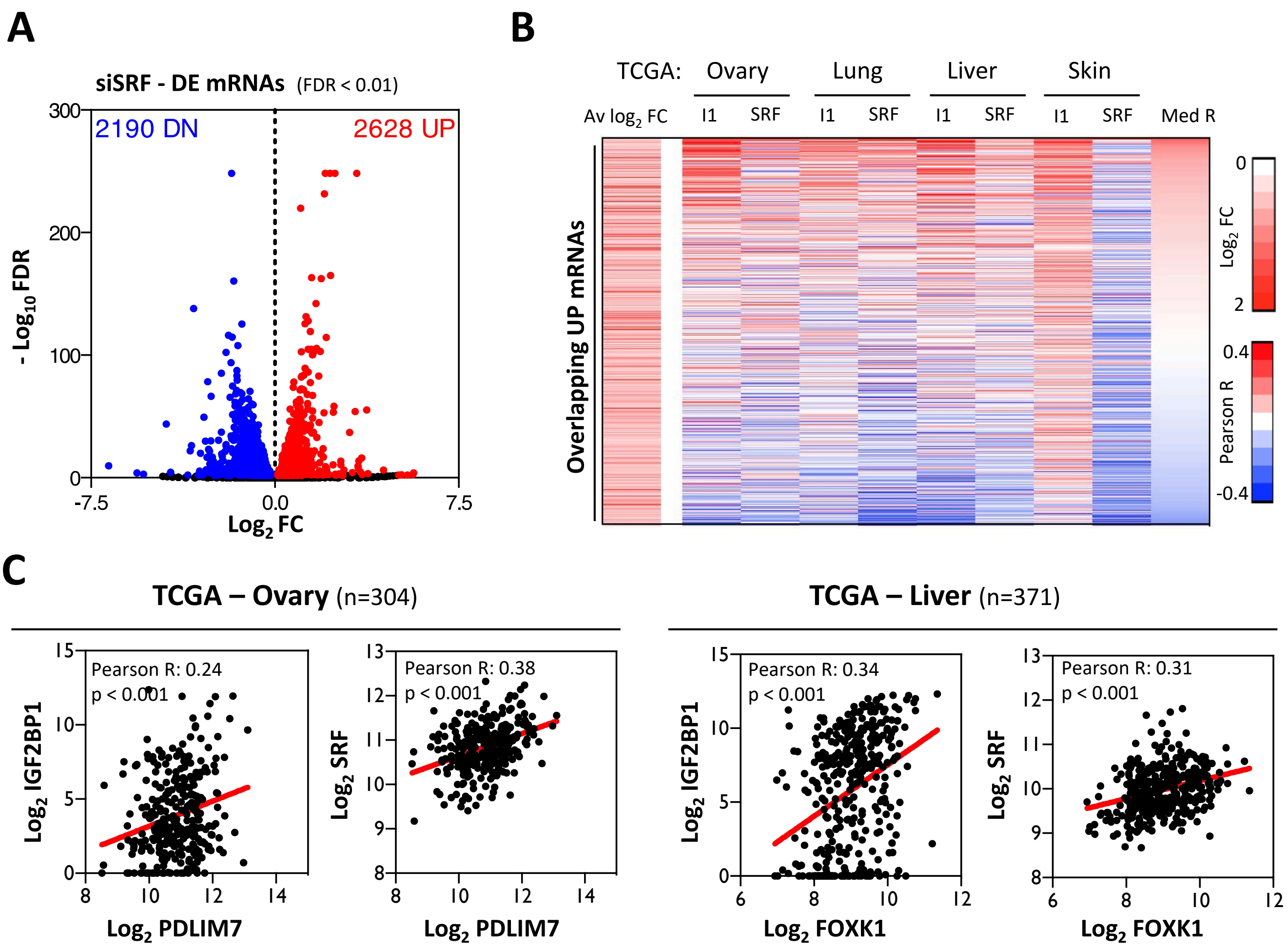

Supplementary Figure 7 – Müller et al.

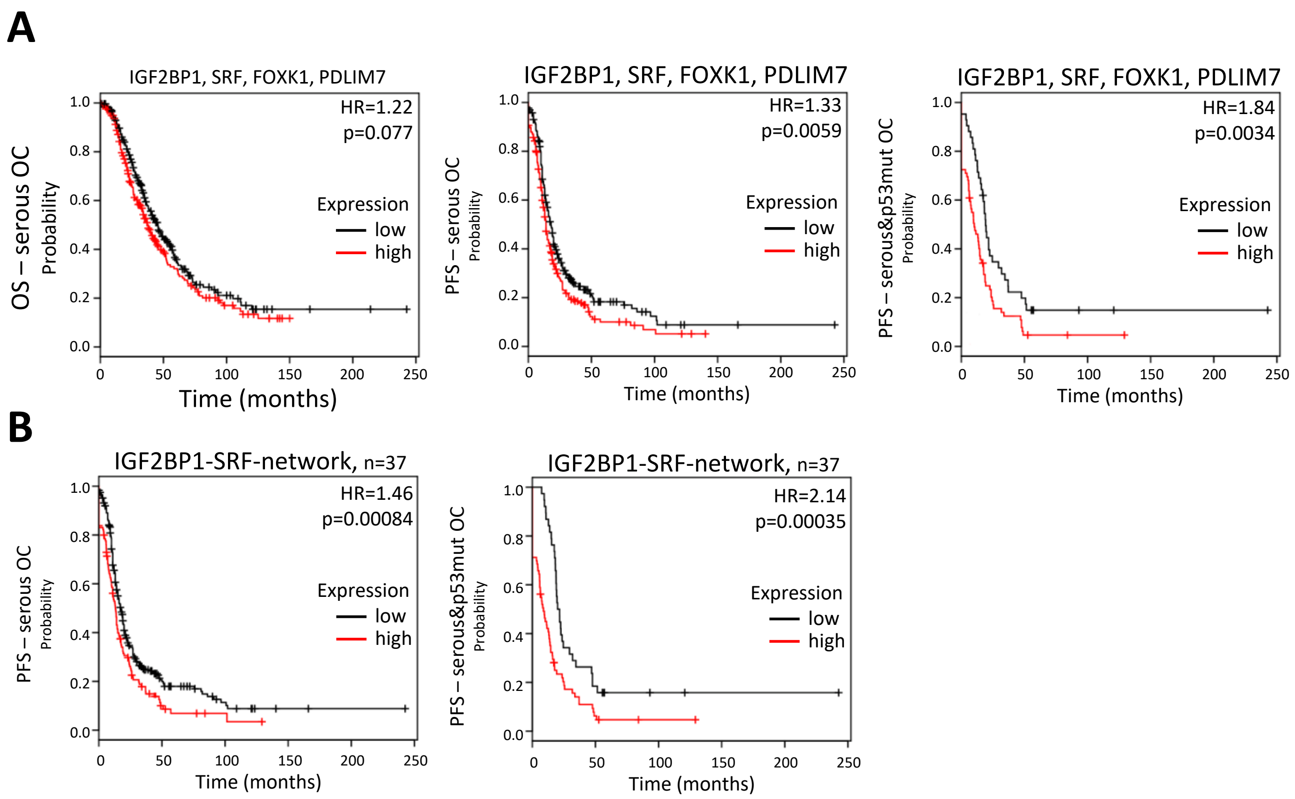

SUPPLEMENTARY FIGURE LEGENDS

Supplementary Figure 1. IGF2BP1-dependent control of gene expression in ES-2 and Huh-7 cells. (**A**) Correlation analysis of miRNA expression, as determined by small RNA-seq (supplementary Table T2), in Huh-7 and ES-2 cells. Downregulated expression of the let-7-5p family (red) and upregulation of miR-122-5p (blue) in Huh-7 cells is indicated along with the determined Correlation coefficient (R), R squared value (R2) and statistical significance (p-value). (**B**) The expression (CPM; counts per million mapped reads) of all nine let-7-5p family members in ES-2 and Huh-7 cells was determined by small RNA-sequencing and summed up over two biological replicates. (**C**) The overlap of mRNAs significantly (FDR ≤ 0.01) up- (UP, red) or downregulated (DN, blue) in the indicated cell lines upon IGF2BP1 depletion is shown by Venn diagrams. Numbers indicate transcripts with significantly deregulated expression in analyzed cell lines. (**D**) The IGF2BP1 3'UTR-CLIP scores determined for mRNAs significantly down- (DN, blue) or upregulated (UP, red) in Huh-7 or ES-2 cell are depicted by box plots. (**E**) Gene set enrichment analysis (GSEA) of IGF2BP1-depleted Huh-7 (top panel) and ES-2 (bottom panel) cells. Genes were ranked according to their fold change. Note that the same pathways are significantly affected by IGF2BP1 depletion (also see supplementary Table 1B). Statistical significance was determined by Student's t-test: (**) $P < 0.01$, (***) $P < 0.001$.

Supplementary Figure 2. (**A**) Knockdown-recovery study indicating that wild type chicken Igf2bp1 restores SRF protein (left panel) and mRNA (right panel) expression in ES-2 cells. RPLP0 served as the internal normalization control in RT-qPCR analyses. Cells stably expressing indicated proteins were previously reported (9). Statistical significance was determined by Student's t-test: (***) $P < 0.001$.

Supplementary Figure 3. IGF2BP1 controls SRF expression in HepG2 cells in an m^6A-dependent manner. (**A**) RT-qPCR analyses demonstrate the downregulation (relative to controls, siC-transfected) of the SRF mRNA upon IGF2BP1 depletion in HepG2 cells by siRNA pools (72h). GAPDH served as the negative and RPLP0 as the normalization control. (**B**) Representative Western blot analysis of indicated proteins in HepG2 cells transfected with control (siC) or IGF2BP1-directed siRNA pools as in A. (**C**) RT-

qPCR analysis showing the upregulation (relative to controls, parental A549 cells) of the SRF mRNA upon deletion of the bulk 3'UTR. GAPDH served as the negative and RPLP0 as the normalization control. Error bars indicate standard deviation determined in at least three analyses. (**D**) The depletion of METTL3 and METTL14 by siRNA pools impairs SRF protein expression in indicated cell lines. GAPDH served as the loading and negative control. Representative Western blots are shown. (**E**) The enlargement of Fig. 2I shows m^6A-RIP-seq reads in the 3'UTR in HepG2 cells transfected with control shRNAs (red) or METTL14-directed shRNAs (blue), as reported by (14). Clip hits in the respective region reported by eCLIP analyses in HepG2 cells are shown in the lower panel. (**F**) IGF2BP1-immunoprecipitation analyzed in Fig. 2J was evaluated by Western blotting in control-transfected (C) or METTL3/14-depleted (M3/14) cells. GAPDH served as the loading control in input fractions. (**G**) Representative Western blot analysis of indicated proteins showing that elevated expression of GFP-tagged wild type IGF2BP1 (I1) restores SRF protein abundance when METTL3 is depleted in ES-2 cells. Note that SRF protein abundance remains unaffected in cells expressing GFP or RNA-binding deficient IGF2BP1 (I1mut). GAPDH served as the loading control. Statistical significance was determined by Student's t-test: (**) $P < 0.01$, (***) $P < 0.001$.

Supplementary Figure 4. IGF2BP1-dependent regulation of SRF expression is IGF2BP paralogue specific. (**A**) Representative Western blot analysis in indicated cell lines demonstrating that only the depletion of IGF2BP1 impairs SRF protein abundance in cancer cells. Cells were transfected with control (siC) or siRNA pools directed against IGF2BP paralogues (IGF2BP1, 2 and 3) for 72h. VCL served as the loading control. (**B**) Schematic indicating the position of CLIP sites reported for IGF2BP1-3 in the SRF 3'UTR by eight (IGF2BP1), seven (IGF2BP2) or six (IGF2BP3) experiments performed in HepG2, K562, HEK293 or hESC cells, as indicated in material and methods. VCL served as the loading control. (**C**) The expression of SRF and IGF2BP1 was tested for Pearson correlation in indicated cancers using TCGA-derived RNA-seq data. The determined correlation coefficients for each analysis as well as the median correlation coefficient (Trend) are indicated by a heat map. Scale bars for R values are shown in the lower panel. (**D**) MiRNA expression was determined by small RNA-seq of two biological

replicates of indicated cell lines (left panel). The CPM of 598 miRNAs predicted by at least 3 of 4 databases (miRWalk, RNA22, TargetScan, miRanda) to target the SRF 3'UTR are shown by a heat map (right panel). MiRNAs are sorted according to the median CPM determined in the analyzed cell lines. Scale bars for CPM values are shown in the lower panel. (**E**) The depletion of DICER and DROSHA, as well as miR-22 upon DICER/DROSHA knockdown in ES-2 cells, was monitored by Western blotting (upper panel, WB) or Northern blotting (lower panel, NB). VCL, 5S rRNA and the U6 snRNA served as loading controls in WB or NB, respectively. Representative blots are shown.

Supplementary Figure 5. (**A**) SRF protein expression was analyzed by Western blotting upon the depletion of IGF2BP1 or ELAVL1 in A549 cells (72 h). Note that only the depletion of IGF2BP1 results in reduced SRF protein abundance. GAPDH served as the loading control. A representative Western blot is shown. (**B**) The relative number of overlapping CLIP sites determined for IGF2BP1, ELAVL1 and AGO2 in the proximity of MTSs, as recently reported (6), are shown relative to the start of MTSs predicted by TargetScan for human mRNAs (hg19; IGF2BP1 all eCLIP) or the SRF 3'UTR (IGF2BP1 SRF eCLIP).

Supplementary Figure 6. Co-regulation of gene expression by IGF2BP1 and SRF in ES-2 cells. (**A**) Volcano plot showing differential gene expression (threshold: FDR ≤ 0.01) determined by RNA-seq in ES-2 cells upon SRF depletion using siRNA pools (72h). (**B**) The expression of genes upregulated by SRF and IGF2BP1 depletion in ES-2 cells was tested for Pearson correlation in indicated cancers using TCGA-derived RNA-seq data. The average log2 fold change (right) of gene expression in ES-2 cells (Av $\log_2$ FC) upon depletion and correlation coefficients (R) determined for IGF2BP1 and SRF in indicated cancers are shown for each upregulated gene by a heat map. Genes are sorted according to the median correlation coefficient (Med R) of gene expression (with SRF and IGF2BP1) indicated on the right. Scale bars for the Av $\log_2$ FC and R are shown in the right panel. (**C**) Pearson correlation analysis of indicated genes in TCGA-derived RNA-seq data of ovarian (ovary) and liver cancer. The number of considered tumor samples, correlation coefficients (R) and statistical significance determined by the R2 database are indicated. Correlations coefficients for all genes analyzed in TCGA-provided datasets (Ovary, Lung,

Liver and Skin) are summarized in supplementary Table T4B.

Supplementary Figure 7. SRF/IGF2BP1-directed gene expression is associated with an unfavorable prognosis in cancer. (**A**) Kaplan Meier analyses of the small gene set (IGF2BP1, SRF, PDLIM7 and FOXK1; see Figure 6G) were performed in an ovarian data set using the multigene classifier of KM plotter. The overall (OS) survival probability in all serous ovarian cancer samples (left panel), the progression-free survival probability (PFS) in all (middle panel) or p53-mutated (right panel) serous ovarian cancer samples accessible via KM plotter are shown. (**B**) Kaplan Meier analyses of the 37 I1-SRF-network genes (see Figure 6G) in serous ovarian cancer (left panel) and p53-mutated serous ovarian cancer (right panel) were performed by KM plotter using the multi gene classifier. The overall (OS) or progression-free (PFS) survival probability along with hazardous ratios (HR) and p values determined by KM plotter are shown.
